# Antioxidant Properties of Unripe* Carica papaya* Fruit Extract and Its Protective Effects against Endothelial Oxidative Stress

**DOI:** 10.1155/2019/4912631

**Published:** 2019-06-20

**Authors:** Wattanased Jarisarapurin, Wariya Sanrattana, Linda Chularojmontri, Khwandow Kunchana, Suvara K. Wattanapitayakul

**Affiliations:** ^1^Department of Pharmacology, Faculty of Medicine, Srinakharinwirot University, Thailand; ^2^Department of Pharmacology, Faculty of Pharmacy, Mahidol University, Bangkok, Thailand; ^3^Department of Clinical Chemistry and Haematology, University Medical Centre Utrecht, Utrecht University, Utrecht, Netherlands; ^4^Department of Preclinical Sciences, Faculty of Medicine, Thammasat University, Khlong Luang, Pathum Thani, Thailand

## Abstract

It has been proven that high consumption of fruit and vegetable lowers the risks of cardiovascular and other oxidative stress-related diseases. Here we evaluated the effects of a tropical fruit, unripe* Carica papaya* (UCP), on endothelial protection against oxidative damage induced by H_2_O_2_. The antioxidant properties of UCP were investigated using the assays of FRAP and ORAC and specific ROS scavenging activities (H_2_O_2_, O_2_^•−^, OH^•^, HOCl). Cytoprotective property was tested in human endothelial cell line EA.hy926 with respect to cell survival, intracellular ROS levels, antioxidant enzyme activities (CAT, SOD, GPX), survival/stress signaling (AKT, JNK, p38), and nuclear signaling (Nrf2, NF-kB). UCP processed high antioxidant activity and scavenging activity against H_2_O_2_> OH^•^> O_2_^•−^> HOCl, respectively. UCP improved cell survival in the milieu of ROS reduction. While SOD was increased by UCP, CAT activity was enhanced when cells were challenged with H_2_O_2_. UCP had no impact on H_2_O_2_-activated AKT, JNK, and p38 signaling but significantly decreased nuclear NF-*κ*B levels. The overactivation of Nrf2 in response to oxidative stress was constrained by UCP. In conclusion, UCP protected endothelial cells against oxidative damage through intracellular ROS reduction, enhanced CAT activity, suppression of NF-kB, and prohibition of Nrf2 dysregulation. Thus, UCP might be a candidate for development of nutraceuticals against CVD and oxidative-related diseases and conditions.

## 1. Introduction

Oxidative stress or elevated levels of ROS constitute a known phenomenon commonly observed in cardiovascular disease (CVD) and vascular aging. ROS are highly reactive and unstable molecules that can scramble electron from various stable molecules in the cells. ROS generated by vascular walls or endothelial cells (ECs), including OH^•^, H_2_O_2_, and O_2_^•−^, play an important role in cell homeostasis and in normal functions involving cell proliferation, survival, migration, and angiogenesis [1]. On the contrary, excessive amount of ROS and/or weakened antioxidant defense can induce ECs, damage, and apoptosis, which are the main causes of endothelial dysfunction [2]. These ROS mediate cellular signaling pathways of inflammatory responses, cell survival, and cell death which involve the development of atherosclerotic lesions that lead to thrombosis and stroke [3]. Given these premises, the rationale for antioxidant therapy is on the horizon, yet the success rate of CVD risk reduction using synthetic antioxidant supplements has been disappointing [4]. Nonetheless, high consumption of dietary antioxidants shows more promising outcomes in both retrospective and prospective clinical studies [5].

In normal condition, intracellular ROS are counterbalanced by two antioxidant systems, i.e., enzymatic and nonenzymatic antioxidants. The enzymatic antioxidants are part of intracellular antioxidant defense system consisting of three major enzymes, including glutathione peroxidase (GPX), superoxide dismutase (SOD), and catalase (CAT). These enzymes are catalytic enzymes that control the amounts of intracellular ROS produced by regular metabolism in cells. When ROS are excess, the major regulator of endogenous enzymatic pool, Nrf2 signaling pathway, is activated and translocated into nucleus, leading to transactivation of ARE that are responsible for enzymatic antioxidant production [6]. On the other hand, nonenzymatic antioxidants can be found in both endogenous and exogenous sources. Endogenous reduced glutathione (GSH) is the limited key reservoir for GPX and other redox homeostasis systems while exogenous supplies of antioxidants are rather extensive, including the well-known antioxidant vitamins (vitamin C, vitamin E, beta-carotene, etc.) and a wide variety of antioxidant compounds from natural sources such as fruits, vegetables, and medicinal plants [7]. Antioxidants have important roles in protecting ECs by decreasing ROS-induced inflammation and cell death, which can reduce risks of endothelial dysfunction and risks of atherosclerosis as well as other CVD [8]. Therefore, protection of ECs from ROS-induced cell death could be a promising strategy to reduce CVD risks.

Due to dissatisfaction with antioxidant therapy by synthetic antioxidant supplements, more emphasis has been placed on dietary and natural sources of antioxidants.* Carica papaya* L. is a dietary plant that is widely grown in tropical and subtropical areas such as Southeast Asia and Mexico. Papaya fruit, also known as papaw or pawpaw, is usually served as a component of meals in many counties, mostly consumed in form of ripe fruits. In Thailand, unripe* Carica papaya* (UCP) is a well-known main ingredient of the popular dish papaya salad. UCP is a good source of antioxidants including vitamin C, gallic acid, terpenoids, alkaloids, flavonoid, and other phenolic compounds [9, 10]. Papaya is one of the natural remedies that have been used in traditional medicine for constipation, wound repair, skin infection, reproductive organ stimulation, and diabetes [11–13]. Previous studies provide the proof for therapeutic effect on wound healing of diabetic rats [14] and antibacterial effect on common wound microorganism [15]. Fermented papaya restored key antioxidant enzymes and protected the oxidative damage of the liver during N-methyl-N-nitrosourea induced hepatocellular carcinoma in Balb/c mice [16]. In Mauritian neo-diabetic subjects, short-term supplement with fermented papaya reduced CVD risk via decreasing inflammation and oxidative stress [17]. Moreover, fermented papaya also decreased the marker of oxidative damage to DNA, i.e., 8-hydroxy-2'-deoxyguanosine (8-OHdG), in patients with Alzheimer's disease [18]. Although the antioxidant and therapeutic effects of papaya on wound healing and some diseases have been reported, the benefit of papaya on ECs has not been mechanistically determined thus far. Therefore, this study focused on antioxidant property of unripe* Carica papaya* fruit (UCP) and its cytoprotective effect on ECs exposed to H_2_O_2_ as a model of oxidative stress. UCP appeared to diminish H_2_O_2_-induced cell death by two main strategies carried out by (1) elimination of intracellular stress (ROS reduction and NF-kB inactivation) and (2) equipping the cells with antioxidant defense (enhanced CAT activity and Nrf2 modification). Therefore, UCP can be a candidate for the development of nutraceuticals for the prevention of oxidative-related conditions such as cardiovascular disease and aging.

## 2. Materials and Methods

### 2.1. Chemicals and Cell Culture Protocol

Chemicals used in this study are analytical or cell culture grades obtained from Sigma-Aldrich (https://www.sigmaaldrich.com/) unless otherwise stated. For experiments using cell culture, the human endothelial cell line EA.hy926 was obtained from ATCC® (CRL-2922™). Cells were grown in DMEM supplemented with 10% FBS, 100 U/mL penicillin, and 100 *μ*g/mL streptomycin in a humidified 5% CO_2_ incubator maintained at 37°C. The cell culture medium was changed every three days until 80-90% confluence. Cell passage was limited to not more than 30th passages. Cells were prepared prior to the beginning of each experiment by seeding in the cultureware and grown for 18-24 h. To evaluate the effect of UCP on H_2_O_2_-induced oxidative stress and cell death, cells were pretreated with various concentrations of UCP (10, 100, 1000 *μ*g/mL) in fresh media for 48 h followed by incubation with 1 mM H_2_O_2_ in fresh media for 2 h.

### 2.2. Preparation of UCP Powder

The UCP fruits were purchased from a fresh market in Bangkok, Thailand, that acquired products consistently from specific farms. UCP fruits were washed to remove dirt and latex; then the fruits were peeled and only flesh was sliced into small pieces. Fresh fruit juice was obtained from a juice extractor and kept on ice throughout the process to protect degradation of the biomolecules and antioxidants. The UCP juice was filtered through sterile qualitative paper (Whatman® grade 1 filter paper) followed by drying into powders by lyophilization technique.

### 2.3. Determinations of Antioxidant Capacity and ROS Scavenging Activity of UCP

#### 2.3.1. FRAP Assay

The reducing power of UCP was evaluated based on the reduction of Fe^3+^ to Fe^2+^ previously described by Benzie et al. [19]. Briefly, the FRAP reagent was prepared by mixing 10 mM TPTZ, 20 mM FeCl_3_ in 40 mM HCl, and 300 mM acetate buffer, pH 3.6, at the ratio of 1:1:10. This reagent was mixed with various concentrations of samples and standards (Fe_2_SO_4_). The reagent was then incubated at room temperature for 5 minutes followed by absorbance detection at 593 nm. FRAP values were calculated from a dose response data plots of sample concentrations (x-axis) and corresponding FeSO_4_ (y-axis) using the linear regression equation y = ax+b. The antioxidant capacity of each sample in this assay was shown as Fe_2_SO_4_ equivalent in *μ*mol per 1 g UCP.

#### 2.3.2. ORAC Assay

This assay measures a fluorescent intensity from AAPH probe that persisted or quenched in the presence of antioxidant or ROS, respectively [20]. Various concentrations of samples were mixed with 10 nM fluorescein in 75 mM KH_2_PO_4_. The mixtures were incubated at 37°C for 5 min. The reaction was started after adding 165 mM AAPH. The fluorescence intensity of fluorescein was measured immediately at the excitation at 485 nm and emission at 528 nm for 60 min with 1-min interval. The area under the curve (AUC) was calculated using the Graphpad Prism software. Net AUC values of each sample were calculated as follows.

(1) $$\begin{array}{c} Net\;AUC=AUC_{sample}-AUC_{blank} \end{array}$$

The ORAC was calculated from a dose response data plots of Net AUC values of sample concentrations (x-axis) and Net AUC values of trolox (y-axis) using linear regression equation y = ax+b. The ORAC of each sample is shown as trolox equivalent (TE) in *μ*mol per 1 g UCP.

#### 2.3.3. OH^•^ Scavenging Activity

The assay was performed as previously described by Mandal et al. [21] with minor modifications. Briefly, the solution mixture containing the final concentration of EDTA (2 mM), FeCl_3_ (0.1 mM), 2-deoxy-2-ribose (1.12 mM), H_2_O_2_ (0.2 mM), and sodium L-ascorbic acid (0.2 mM) was mixed with various concentrations of UCP and standard (trolox) dissolved in 0.1 M KH_2_PO_4_ buffer, pH 7.4. The reactions were continued at 50°C for 20 minutes, and then TCA and TBA (1.12% and 0.4% final concentration, respectively) were added and further incubated at 95°C for 15 minutes. The mixtures were cooled down, and the absorbance of solutions (As) and blank (no samples or no standards added, A_B_) was measured at 550 nm. The percent inhibition of each sample and IC50 were calculated as follows.

(2) %  inhibition=AB−ASAB×100IC50=50−ba

IC50 values were calculated from a dose response data plots of sample concentrations (x-axis) and %inhibition (y-axis) using the linear regression equation y = ax+b. This formulation was used for determination of IC50s of the rest of ROS scavenging activity assays.

#### 2.3.4. HOCl Scavenging Activity

The assay monitors the chromogen 5-thio-2-nitrobenzoic acid (TNB) remaining that inhibits oxidation reaction with HOCl in the presence of scavenging activity of antioxidants, according to Valentao P et al. [22]. Briefly, 40 *μ*M of HOCl and 40 *μ*M of TNB were prepared and diluted to 1 mM with 50 mM KH_2_PO_4_, pH 6.0, containing 5 mM EDTA. The experiment was initiated by mixing with various concentrations of samples and standards (ascorbic acid) with 40 *μ*M TNB, and the absorbance was measured at 412 nm before (A_before_) and 5 min after (A_after_) adding 40 *μ*M HOCl using a spectrophotometer. The percent TNB remaining in each sample and IC50 were calculated as follows.

(3) %  TNB  remaining=100−Abefore−AafterAbefore×100

#### 2.3.5. Superoxide Anion Radical (*O*_2_^•−^) Scavenging Activity of UCP

The O_2_^•−^ scavenging activity of UCP was evaluated by the modified assay based on the method described by Kumar R. et al. [23]. The reaction mixture, containing 77.4 *μ*M NBT and 90 *μ*M NADH in 19 mM KH_2_PO_4_ buffer, pH 7.4, was made with various concentrations of samples and standards. The reactions were started by adding PMS dissolved in 19 mM KH_2_PO_4_ buffer, pH 7.4, to the final concentration of 9 *μ*M and then incubated at room temperature for 3 min. The O_2_^•−^ was produced by the reaction of PMS and NADPH, which converts NBT to NBT formazan. The formazan formation was monitored at 560 nm using a spectrophotometer. The absorbance of solutions (As) and blank (no samples or standards added, A_B_) was used for the calculation of percent inhibition and IC50 as follows.

(4) $$\begin{array}{c} \%\;inhibition=\left[\;\frac{\left(A_{B}-A_{S}\right)}{A_{B}}\;\right]\times 100 \end{array}$$

#### 2.3.6. H_2_O_2_ Scavenging Activity of UCP

The measurement of H_2_O_2_ scavenging activity followed the method described by Paital et al. [24]. The reaction mixture containing the final concentrations of 125 *μ*M of homovanillic acid (HVA) and 0.1 U of HRP dissolved in 50 mM KH_2_PO_4_ buffer, pH 7.4, was made with the various concentrations of sample and standard (trolox) in 96-well plate. The reaction was started by adding 30 *μ*M H_2_O_2_ and incubated at room temperature for 30 min. HVA reacted with HRP to generate fluorescence HVA dimer, which was measured at excitation and emission absorbance of 315 and 425 nm, respectively. The absorbance of solutions (As) and blank (no samples or standards added, A_B_) was monitored and used for the calculation of percent inhibition and IC50 of each sample, as follows.

(5) $$\begin{array}{c} \%\;inhibition=\left[\;\frac{\left(A_{B}-A_{S}\right)}{A_{B}}\;\right]\times 100 \end{array}$$

### 2.4. Cell Viability Assay

The protective effect of UCP on H_2_O_2_-induced EA.hy926 cell death was determined by MTT cell viability assay as described previously [25]. Briefly, cells were seeded in 96-well plate at a density of 5 x 10^3^ cells/well for 18-24 h. The fresh media containing various concentrations of UCP (10, 100, 1000 *μ*g/mL) were preincubated in each well for 48 h. After incubation, the culture media were removed and replaced with 1 mM H_2_O_2_ for 2 h. The supernatant was removed, and cells were incubated with 0.25 mg/mL MTT in DMEM medium for 3 h. The levels of DMSO-dissolved formazan were measured at 550 nm using spectrophotometer (SpectraMax M2e). Data are shown as the percentage of cell viability compared with vehicle treated group.

### 2.5. Measurement of Intracellular ROS

The intracellular ROS of EA.hy926 cells were evaluated by flow cytometry using DCFH-DA probe. Briefly, 1.5 x 10^5^ cells were seeded in 60-mm cell culture dish and incubated for 18-24 h. The various concentrations of UCP (10, 100, 1000 *μ*g/mL) were pretreated in each dish for 48 h. The media were removed and replaced with 1 mM H_2_O_2_ for 2 h. The cells were washed with 1 x PBS twice and replaced with 25 *μ*g/mL DCFH-DA dissolved in fresh media, and further incubated for 30 min. Then, cells were washed with 1 x PBS twice and collected by trypsinization. Following two washes, cell concentration was adjusted to 500 cells/*μ*L with 1 x cold PBS. Intracellular ROS of each cell were evaluated by measuring the fluorescence intensity using flow cytometer (Millipore/Guava® easyCyte™ 8 HT). The unstained cell population was used to determine baseline ROS range. The percentage of mean fluorescent intensity was used to evaluate the intracellular ROS using control as a reference (100%).

### 2.6. Measurement of Apoptotic Cell Death

The apoptotic cells were evaluated by Hoechst and propidium iodide (PI) staining. The cells were seeded in each 35 mm cell culture dish at the density of 5 x 10^4^ cells/dish and incubated for 18-24 h. Following UCP preincubation for 48 h, 1 mM H_2_O_2_ was replaced, and then the cells were further incubated for 4 h. The cells were washed with 1 x PBS twice and incubated with 1 *μ*g/ mL Hoechst in phenol red free DMEM for 15 minutes. After a couple of washes with 1x PBS, cells were incubated with 1 *μ*g/ mL PI in phenol red free DMEM for 15 min. Cells were then washed and replaced with 1 x PBS. Several photos depicting fluorescent nuclei were captured under fluorescence microscope (Olympus). To avoid bias, the apoptotic and normal cells were distinguished and evaluated by the color threshold application of the Image J software. The data are presented as the percentage of apoptotic cells compared with control group, as in the following equation.

(6) $$\begin{array}{c} \%\;Apoptotic\;cell=\left[\;\frac{\left(Apoptotic\;cells\right)}{\left(Total\;cells\right)}\;\right]\times 100 \end{array}$$

### 2.7. Measurement of GPX Activity

This assay determined GPX activity from the reduction of NADPH, which converts GSSG to GSH and cooperates with glutathione reductase (GR) in GPX cycle, as described by Weydert and Cullen [23]. Briefly, cells were seeded in 60 mm dish at a density of 2 x 10^5^ cells/dish and incubated for 18-24 h. Following UCP and H_2_O_2_ treatments, cells were washed with 1 x cold PBS twice and collected by cell scrapper. After sonication, the supernatants were collected and kept at -80°C until use. To initiate the GPX cycle, 20 *μ*L of samples were added in 96-well plate followed by 100 *μ*L of GPX assay buffer (50 mM Tris buffer pH 7.4 containing with 1 mM EDTA). The 50 *μ*L of co-substance mixer containing 0.6 mg/mL of NADPH, 0.4 mg/mL of GSH, and 5 units/mL of GR was added in each well and placed on a shaker. The reaction was started by adding 20 *μ*L of 15 mM cumene hydroperoxide, and the absorbance was quickly measured at 340 nm every 1 minute for 1 hour. The GPX activity (nmol/min/mL/mg protein) was calculated using the following equations.

(7) ΔA340/min=ΔA340Time2−ΔA340Time1Time2−Time1GPX  activitynmol/min/mL=ΔA340/min×Total  volume×Dilution  factorNADPH  extinction  coefficient×Sample  volume

### 2.8. Measurement of SOD Activity

SOD assay kit (Sigma Cat. No. 19160) was used to evaluate SOD activity. Briefly, cells were seeded in 60 mm dish at a density of 2 x 10^5^ cells/dish and incubated for 18-24 h. The cells were treated and protein lysates were collected as described in GPX assay. Then, the assay was performed following the manufacturer's instructions. SOD activity of each sample was presented as nmol/min/mL.

### 2.9. Measurement of CAT Activity

The assay is based on the reaction between methanol and H_2_O_2_ catalyzed by CAT to produce formaldehyde which interacts with Purpald® chromogen to generate purple colored complex [26]. Cells were treated and protein lysates were collected as described in GPX method. 20 *μ*L of various concentrations (0-120 *μ*M) of standard formaldehyde and lysate samples were prepared in sample buffer (100 mM KH_2_PO_4_ buffer, pH 7.5, containing 1mM EDTA and 0.1% BSA). Then, 100 *μ*L of test buffer (sample buffer + 30% methanol) was mixed in each well, and then the reaction was started by adding 20 *μ*L of 35 mM H_2_O_2_ in distilled water and incubated for 20 min. The amounts of formaldehyde formation were detected by adding 50 *μ*L of 3 mg/mL Purpald® chromogen in 0.5 M KOH and incubated for 10 min on a shaker. Then 10 *μ*L of 65.2 mM potassium periodate in 0.5 M KOH was added and incubated for 5 minutes on a shaker. The absorbance of oxidation product was measured at 540 nm (SpectraMax M2e). The CAT activity was calculated by extrapolation of formaldehyde standard curve as follows.

(8) FormadehydeμM=(As−y−interceptSlope×(Total−Vs0.17 mL(Vs0.02 mLCAT  activitynM/min/mL=FμMReaction  time20 min×Dilution  factor

*Note*: As is the sample absorbance; Vs is the sample volume; and F is the formaldehyde concentration of the sample.

### 2.10. GSH Measurement

Total GSH levels were determined as previously described [27]. Briefly, the GSH standard (0.5-20 *μ*M) and protein lysates were diluted in 100 mM phosphate buffer containing 1 mM EDTA, pH 7.4 (assay buffer). Next, equal amount of 1% metaphosphoric was added to each well of 96 well plate. Then, 50 *μ*L of 0.5mg/mL DTNB in assay buffer containing 1 mg/mL BSA was added to each well. The reaction was initiated by mixing the reaction with GSH reductase (1 Unit/mL) and 50 *μ*L of 0.3 mg/mL NADPH dissolved in assay buffer. The GSH content is monitored at absorbance at 415 nm.

### 2.11. Western Blot Analysis

Proteins from lysates were collected in total and nuclear fractions. Cells were seeded in 60 mm cell culture dish at density of 2 x 10^5^ cells/dish incubated for 18-24 h. Cell lysate was prepared in RIPA buffer containing protease inhibitors and phosphatase inhibitors. The nuclear lysate was collected using nuclear extraction kit (Cayman No. 10009277). The protein concentration of each lysate was determined using Bio-Rad protein assay referring to standard BSA. Equal amount of each sample was separated by the SDS-PAGE and transferred to PVDF blotting membrane (Amersham™ Hybond™ 10600023) using Mini-PROTEAN Tetra system (Bio-Rad). Blotted membrane was blocked in blocking solution (5% BSA or nonfat-dry milk) for 1 hour and incubated with primary antibody (1:1000 phospho-AKT, 1:1000 AKT, 1:1000 phospho-JNK, 1:1000 JNK, 1:1000 phospho-p38, 1:1000 p38, 1:1000 Nrf2, 1:1000 NF-kB, or 1:3000 beta-actin) overnight. The membranes were washed with 1 x Tris-buffered saline containing 1% tween (TBST) three times and incubated with 1:3000 anti-rabbit IgG or anti-mouse IgG, HRP-linked antibody for 1 hour. Then the membrane was washed with 1 X TBST three times. The protein bands were visualized using the ECL western blot reagent (Amersham™ ECL Select™) and the illuminating bands were recorded using gel documentation system (UVITEC, Cambridge, United Kingdom). The quantification of protein bands was analyzed using ImageJ software (https://imagej.net/ImageJ1).

### 2.12. Measurement of Nrf2 Transcription Factor by ELISA

The amount of Nrf2 transcription factor was determined by Nrf2 transcription factor assay kit (Cayman No. 600590). The assay procedures were performed according to the manufacturer's instructions. Data are presented as the relative percentage for the absorbance at 450 nm compared with control group.

### 2.13. Statistical Analysis

In this study, each experiment was performed at least three times. Data are shown as mean ± SEM and analyzed by one-way ANOVA. The statistical significance was set at the p values less than 0.05.

## 3. Results

### 3.1. UCP Powder

**Figure 1:**
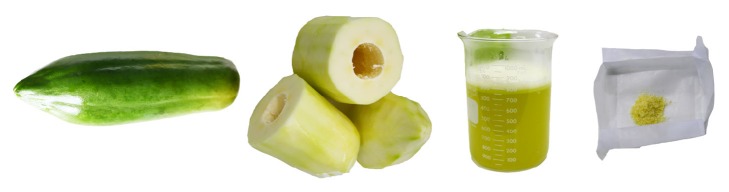
UCP freeze-dried powder process.

Shown in Figure 1 is UCP dry power obtained at the yield of 1.18% (w/w) or 3.6% (w/v). The dry powders were stored at –40°C until use. Prior to performing each experiment, the dry powders were freshly dissolved in distilled water to make a stock solution at 10 mg/mL.

### 3.2. Determination of Antioxidant Potential of UCP

**Figure 2:**
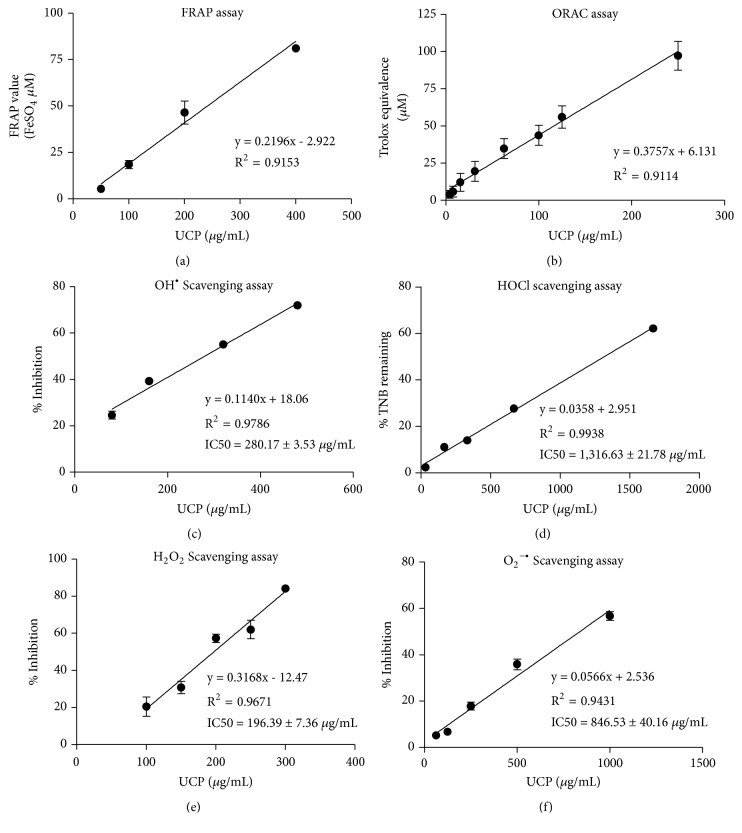
The linear regression of total antioxidant scavenging capacity and specific ROS scavenging activities. Five concentrations of UCP were tested, and linear regression lines showing the correlation between UCP concentrations (x-axis) and indicated y-axis were generated as described in Materials and Methods. (a) FRAP assay; (b) ORAC assay; (c) hydroxyl radical assay; (d) hypochlorous assay; (e) hydrogen peroxide assay; (f) superoxide assay.

The total antioxidant capacity and scavenging activity assays showed that UCP inhibited ROS in a dose-dependent manner (Table 1). The linear relationships were observed all across antioxidant testing (Figure 2). The ROS scavenging activities of UCP were ranked in order from high to low as follows: H_2_O_2_>OH^•^ > O_2_^•−^ > HOCl.

### 3.3. Effect of UCP on the Cell Viability of H_2_O_2_-Induced EA.hy926 Cell Death

**Figure 3:**
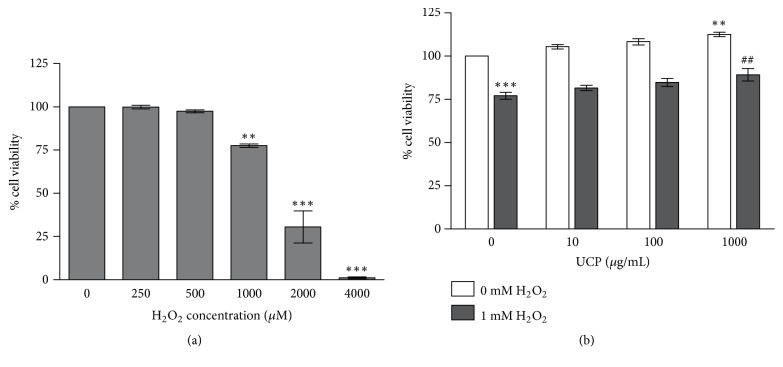
The protective effect of UCP on H_2_O_2_-induced EA.hy926 cell death evaluated by MTT assay. Cell viability was evaluated by MTT assay as described in Materials and Methods. (a) Effects of H_2_O_2_ on cell viability when incubated with various concentrations of H_2_O_2_ (250-4000 *μ*M) for 2 h. (b) Effects of UCP on cell viability in the presence of H_2_O_2_. Data are presented as mean ± SEM. *∗∗* p < 0.01, *∗∗∗* p < 0.001 when compared with vehicle treated group; ## p < 0.01 when compared with 1 mM H_2_O_2_ treated group.

MTT assay showed that H_2_O_2_ at the concentrations lower than 1 mM did not change cell survival whereas H_2_O_2_ at concentrations of 1, 2, and 4 mM significantly decreased cell viability to 77.67 ± 1.02%, 30.57 ± 9.32%, and 1.08 ± 0.5% when compared with vehicle treated group (Figure 3(a)). Endothelial cells pretreated with 1000 *μ*g/mL UCP for 48 h before 1 mM H_2_O_2_ challenge for 2 h showed significant improve in cell viability to 91.86 ± 4.26 % (p < 0.05) when compared with H_2_O_2_ treated group (76.99 ± 2.00%) (Figure 3(b)).

### 3.4. Effect of UCP on H_2_O_2_-Induced EA.hy926 Apoptosis

**Figure 4:**
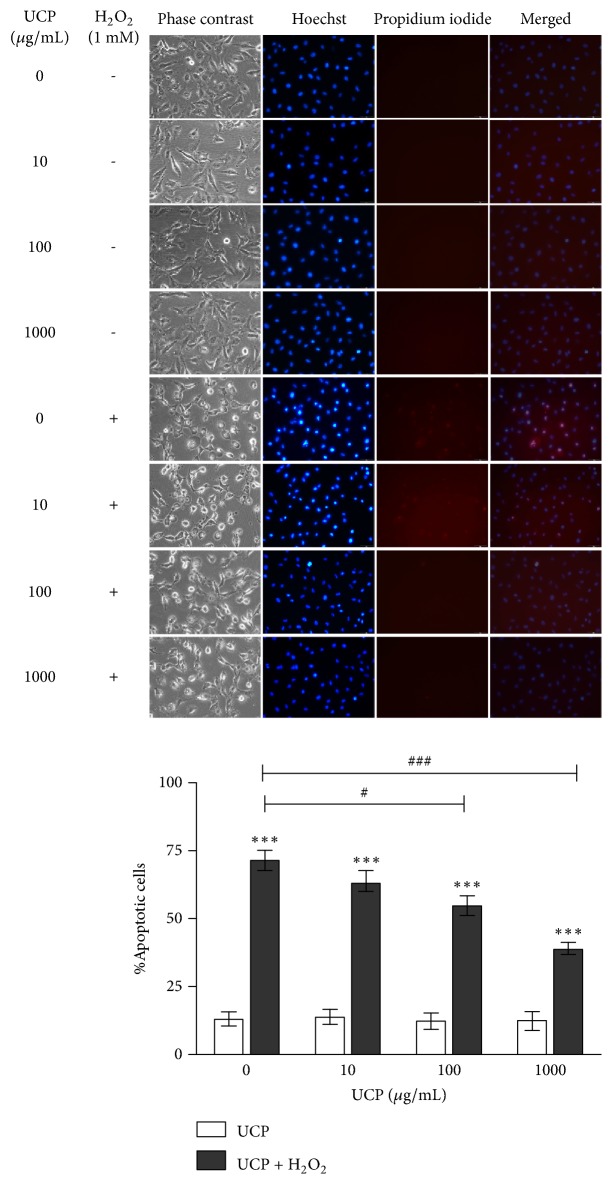
The Hoechst and PI staining of H_2_O_2_-induced EA.hy926 cell death. Cells were pretreated with UCP for 48 h and later exposed to 1 mM H_2_O_2_ for 4 h as described in Materials and Methods. Data are presented as mean ± SEM. *∗∗∗* p < 0.001 compared with control group. # p < 0.05 and ### p < 0.001 compared with 1 mM H_2_O_2_ treated group.

H_2_O_2_ significantly increased the percentage of apoptotic cells to 71.74 ± 3.68% when compared with vehicle treated group (12.91 ± 2.25 %). On the other hand, UCP pretreatment at concentration 100 and 1000 *μ*g/mL significantly decreased number of H_2_O_2_-induced apoptotic cells to 54.84 ± 3.6% and 39.06 ± 2.09% at p < 0.05 and p < 0.001, respectively (Figure 4). These results suggested that UCP pretreatment before exposure to 1 mM H_2_O_2_ had protective effect against H_2_O_2_-induced EA.hy926 cell death.

### 3.5. Effect of UCP on Intracellular ROS

**Figure 5:**
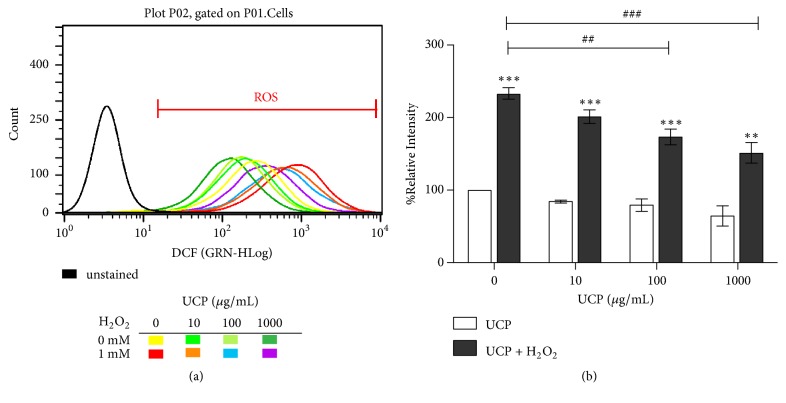
Evaluation of UCP effect on intracellular ROS of EA.hy926 cells using flow cytometric analysis. (a) Histogram plot of unstained sample compared with florescent intensities of cells treated with UCP and/or H_2_O_2_. (b) Graph represents calculation of % relative DCFH-DA intensity of the data in graph (a). *∗∗∗* p < 0.001 compared with vehicle treated group. ## p < 0.01 and ### p < 0.001 compared with 1 mM H_2_O_2_ treated group.

UCP dose-dependently lowered intracellular ROS in EA.hy926 cells as determined by flow cytometric assay using DCFH-DA probe. H_2_O_2_ treatment increased the percentage of mean intensity of DCFH-DA green fluorescence to 233.04 ± 7.56% (p < 0.001) when compared with vehicle treated group. Cells pretreated with 100 and 1000 *μ*g/mL of UCP before 1 mM H_2_O_2_ exposure showed significant decreases in the percentage of mean intensity to 173.53 ± 10.68 and 151.18 ± 14.68 % at p < 0.01 and p<0.001, respectively (Figure 5).

### 3.6. Effect of UCP on Endogenous Antioxidant Enzyme Activities and GSH Levels

**Figure 6:**
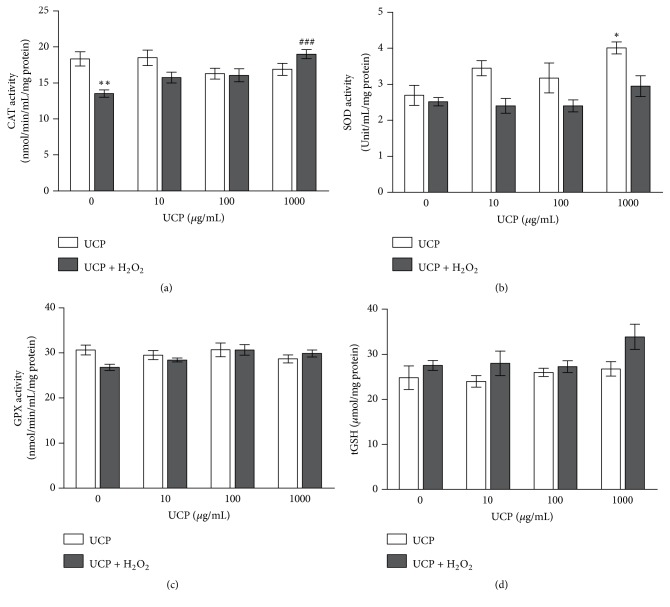
The effect of H_2_O_2_ and UCP on antioxidant enzyme activity and GSH levels in EA.hy926. (a) CAT activity; (b) SOD activity; (c) GPX activity; (d) GSH levels. Data are shown as mean ± SEM. *∗* p < 0.05, *∗∗* p<0.01, and *∗∗∗* p<0.001 when compared with vehicle treated group. ### p < 0.001 when compared with 1 mM H_2_O_2_ treated group.

- CAT activity: H_2_O_2_ treated cells showed significant decrease in CAT activity by 26% (from 18.34±1.01 to 13.53±0.52 mmol/min/mL/mg protein, p < 0.01). UCP pretreatment at the concentration of 1000 *μ*g/mL before exposure to 1 mM H_2_O_2_ showed significant increase in CAT activity to 19.01 ± 0.62 when compared with H_2_O_2_-treated group (p<0.001) (40.5% increase) (Figure 6(a)).
- SOD activity: Decreases of SOD activity were observed in all cell lysate samples treated with H_2_O_2_, yet they did not reach statistical significance. Only the sample from UCP (1000 *μ*g/mL) treated cells alone enhanced SOD activity by 49% (UCP, 2.7±0.28 versus control, 4.01±0.16 Unit/mL/mg protein) (Figure 6(b)).
- GPX activity: H_2_O_2_ treated cells showed no significant decrease in GPX activity when compared with vehicle treated group (30.65±1.1. versus 26.81±0.68 mmol/min/mL/mg protein, respectively). UCP also had no impact on GPX activity whether alone or in combination with H_2_O_2_ treatment (Figure 6(c)).
- Total GSH levels: Neither H_2_O_2_ exposure nor UCP treatment altered GSH contents in EA.hy926 cells (Figure 6(d)).

### 3.7. Effect of UCP on the Signaling of AKT, JNK, and p38

**Figure 7:**
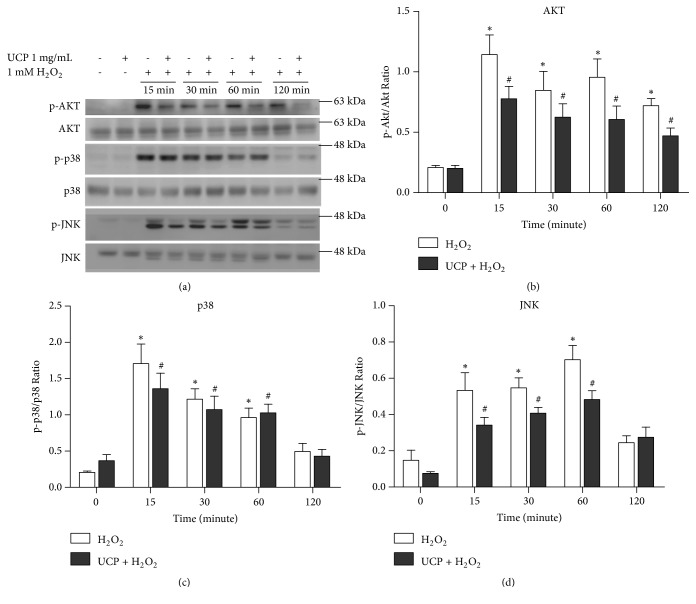
The survival and stress-activated protein kinases response to H_2_O_2_ and/or UCP treatment in EA.hy926. (a) Representative protein bands were determined by western blot analysis. (b) The calculated protein ratio of p-AKT/AKT. (c) The calculated protein ratio of p-p38/p38. (d) The calculated protein ratio of p-JNK/JNK. Data are presented as mean ± SEM. *∗* p < 0.05 and # p < 0.05 when compared to control of the same series data on the graphs.

H_2_O_2_ activation of survival and death signaling proteins including AKT, p38, and JNK was observed for 120-min time intervals (Figure 7(a)). Following 1 mM H_2_O_2_ exposure, phosphorylation of AKT (p-AKT) was timely declined until 120 min. UCP (1 mg/mL) preincubation appeared to decrease AKT activation but did not meet the criteria of statistical significance (Figure 7(b)). Similarly, detection of p-p38 was found to descend within 2 h without any influence of UCP preincubation (Figure 7(c)). For JNK signaling, increased phosphorylation was detected at 15 min and peaked at 60 min prior to a downfall at 2 h. UCP turned down JNK phosphorylation but it did not meet statistically significant difference (Figure 7(d)).

### 3.8. Effect of UCP on Nuclear Signaling of NF-*κ*B and Nrf2

**Figure 8:**
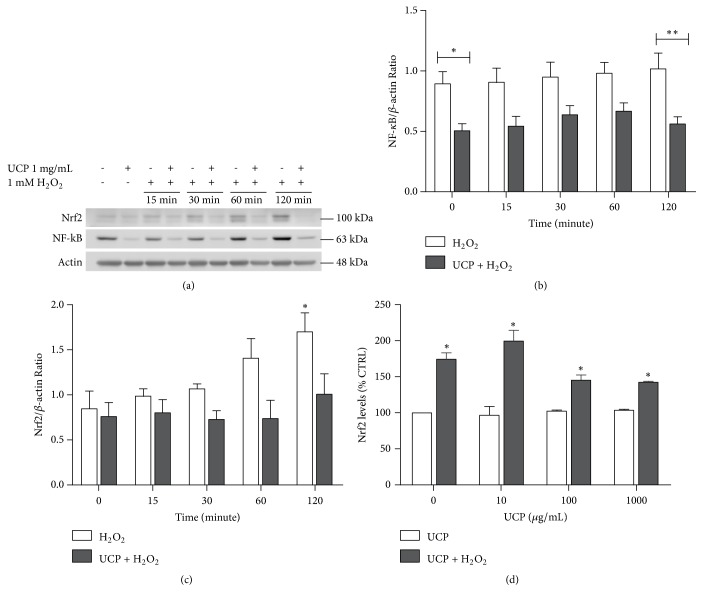
The effect of UCP and H_2_O_2_ on the nuclear transcription factor Nrf2 and NF-*κ*B levels. (a) Representative western blot protein bands at the time frame of 0 to 120 min. (b) Calculated nuclear NF-*κ*B/*β*-actin protein ratios. (c) The Nrf2/ *β*-actin ratio. (d) The amounts of Nrf2 protein determined by ELISA. Data are presented as mean ± SEM. *∗* p < 0.05 and *∗∗* p < 0.01 when compared with vehicle treated group (CTRL).

The nuclear presence of NF-*κ*B and Nrf2 protein was determined by western blot analysis (Figure 8(a)). At basal level, UCP significantly lessened NF-*κ*B translocation to the nuclei of EA.hy926 cells. This trend was maintained until 120 min after H_2_O_2_ exposure (Figure 8(b)). Surprisingly, the amounts of transcription factor Nrf2 were elevated by H_2_O_2_ challenge and showed significance at 120 min while UCP sustained Nrf2 levels all along the time frame of detection (Figure 8(c)). The determination of protein levels of Nrf2 was confirmed again by ELISA assay at 2 h after H_2_O_2_ exposure. Consistently, elevated quantity of Nrf2 in nuclear extract of samples from H_2_O_2_ treated cells was detected whereas 48 h incubation with different concentrations of UCP did not alter nuclear Nrf2 levels (Figure 8(d)).

## 4. Discussion

ROS are regularly generated from cellular metabolism of the living cells such as mitochondrial respiratory chain reactions. In normal condition, ROS or oxidants play significant roles in cellular homeostasis and redox signaling in both continual physiological and aging processes [28, 29]. In endothelial cells, oxidative stress can cause cellular dysfunction and cell death that lead to atherosclerotic plague formation and ultimately CVD [30]. The model of H_2_O_2_-induced endothelial cells oxidative stress used in this study mimics the prominent role of H_2_O_2_ in the regulation of endothelial function along with its induction of inflammation and apoptosis [31]. Here we demonstrate that the natural source of antioxidants, UCP, possessed high antioxidant capacity and scavenging activities against H_2_O_2_>OH^•^>O_2_^•−^>HOCl, in the order of high to low, respectively. UCP ameliorated H_2_O_2_-induced cell death through the mechanisms involving antioxidant/scavenging activities, intracellular ROS reduction, promotion of CAT activity, modification of Nrf2 activity, and abrogation of NF-kB nuclear signaling. ORAC and FRAP are widely used methods to evaluate antioxidant capacity of fruits and vegetables since the results can be compared across laboratories [32]. The antioxidant capacity of UCP in 1 gram dry weight is comparable to 2 mg of blackcurrant and black carrot in form of purified anthocyanin sample (PAS) [33]. The fresh extraction of UCP without solvents and heat-related processes as well as its highly concentrated dry weight (1.18% w/w) may account for high antioxidant capacity of this sample. While ORAC is commonly used as reference for comparing antioxidant capacity of natural products from the industries, FRAP values are often integrated in dietary antioxidant index/capacity for clinical studies. For instance, Mancini et al. [34] reported linear association between risk reduction of type 2 diabetes and consumption of high FRAP diet up to 15 mmol/day which is comparable to UCP approximately 558 g dry powder or 47.3 kg fresh fruit. These data suggest that a practical approach to reaching the maximum FRAP value intake per day is to consume combinations of UCP and other high antioxidant diets.

In addition to total antioxidant capacity, specific scavenging activities against H_2_O_2_, OH^•^, O_2_^•−^, and HOCl reveal its potential use in certain settings and conditions. Many ROS are interrelated or generated by the transformation from one to another. For example, the conversion of O_2_^•−^ by SOD generates H_2_O_2_ which is then transformed to H_2_O by intracellular antioxidant enzymes peroxiredoxins (PRX), GPX, and CAT. Exaggeration of H_2_O_2_ production increases the generation of OH^•^ by Fenton reaction [35]. Among these important ROS, UCP showed relatively highest scavenging activity against H_2_O_2_ which plays a central role as a mediator of ROS conversion to another ROS. H_2_O_2_ also plays an important role in the impairment of redox signaling through the oxidation of cysteine residues within proteins. The uncontrolled levels of H_2_O_2_ beyond partaking in normal physiological function, particularly in endothelial cells, can lead to apoptotic cell death [31]. Therefore, the high antioxidant potential of UCP may diminish oxidative stress and cell death, partly, through the reduction of the central mediator H_2_O_2_. Although UCP had relatively lowest activity in scavenging HOCl, its possible role in modulation of myeloperoxidase-induced cell damage in inflammatory diseases, such as neurodegenerative disease and atherosclerosis, requires further investigations.

Even though it is not possible to directly postulate the* in vitro* chemical-based antioxidant activity of UCP onto* in vivo* milieu, we observed a remarkable reduction (82%) of intracellular ROS in endothelial cells exposed to H_2_O_2_ alongside the downturn of Hoechst-positive apoptotic cell population. Because H_2_O_2_ was the main ROS insult in the model of this study, SOD has minimal involvement in removal of the oxidative stress. UCP alone upregulated SOD activity may be beneficial in other circumstances where O_2_^•−^ is dependable as in mitochondrial oxidative stress implicated in aging-associated CVD [36]. The responses of endogenous antioxidant enzyme defense suggested that, rather than GPX (and its cosubstrate, GSH), CAT played a pivotal role in eliminating exogenous high concentration H_2_O_2_, which is consistent with previous report by Makino et al. [37]. UCP may provide flourishing antioxidant environment that can reduce oxidative stress, partly, by restoring CAT activity in H_2_O_2_-challenged endothelial cells. This phenomenon may resemble the experiment in yeast in which CAT activity was enhanced due to the adaptation to sublethal H_2_O_2_ concentrations only in nutrient-rich media but not in buffer [38]. Correspondingly, insertion of peroxisomal CAT into streptozotocin-induced diabetic C57Bl/6 mice or human retinal cells reduced H_2_O_2_-induced oxidative damage [39]. The impaired CAT and GPX function was also evident in aging sarcopenia where there is a loss of endogenous enzymatic antioxidant protection against elevated levels of H_2_O_2_ in skeletal muscle of aged mice [40]. The deteriorated CAT activity was related to oxidative stress found in renal proximal tubular epithelial cells from spontaneously hypertensive rats (GPX activity was intact) [41] as well as in children with *α*-1 antitrypsin deficiency [42]. In some measure, CAT intervention may undermine oxidative stress-induced pathophysiology or aging process.

In H_2_O_2_-induced EA.hy926 cell death model, UCP showed its protective effect by decreasing intracellular ROS involved in apoptotic pathways. The high loading of H_2_O_2_ to the cells could evoke mitochondrial ROS release (ROS-induced ROS release) and trigger or modify downstream signaling pathways associated with stress-activated protein kinases (SAPK/JNK, p38), inflammation (NF-kB), cell survival (PI3K/AKT), and endogenous antioxidant defense (Nrf2/ARE) [43]. H_2_O_2_ rapidly stimulated AKT, JNK, and p38 phosphorylations; however, UCP did not have considerable influences on this signaling although subtle reductions were observed. It is necessary to take into account other pathways that orchestrate the ultimate outcome of cell fate. This is rather presuming that UCP tended to protect cells death by two main mechanisms. First, UCP minimized the degree of intracellular oxidative stress by the reduction of ROS through scavenging ROS and activation of CAT. Second, UCP inhibited endothelial cells predisposition to the activation of the inflammatory and apoptotic signal NF-kB prior to H_2_O_2_ exposure and the signal was held afterwards. The deviating early upregulation of Nrf2 was also constrained by UCP.

Oxidative damage often occurs when the first line antioxidant defense (i.e., SOD, CAT, GPX) is defeated by the overwhelming ROS, concomitant with subsequent delinquency of the second (antioxidant molecules), third (repair enzymes), and fourth line (antioxidant homeostasis) defenses [44]. The major two arms of responses to biological redox reactions are (1) antioxidant response to oxidative stress and (2) alterations of redox signaling, in which these two arms are interrelated [45]. In the condition of cardiac hypoxia due to ischemia, Nrf2 and NF-kB activation are the primary molecular mechanisms responsible for oxidative modifications as well as proinflammatory effects in cardiac and vascular tissue [46]. The delay or deficiency of antioxidant responses to oxidative stress can result in ER stress, disrupted Nrf2 pathway, and suppressed SOD function as demonstrated in the models of ischemia reperfusion of cerebral cortex and Alzheimer's disease [47, 48]. Nrf2 activation is mainly regulated by its regulatory binding protein Keap1. Oxidative stress induces dissociation of the inactive Nrf2/Keap1 complex, leading to the translocation of Nrf2 to the nucleus and activation of antioxidant defense genes. Early overactivation of Nrf2 may result in the loss of oxidative defense, leading to pathological damage shown in these two distinct models of oxidative stress. The ultimate unifying SOD dysfunction in both models of studies mentioned above substantiates the regulation of posttranslational modifications of Nrf2 pathway in a time-dependent and a cell restricted pathway. Early and aggressive activation of Nrf2 may lead to the expression of antioxidant enzymes as an adaptive response to oxidative stress but could subsequently prompt depletion of endogenous defense and eventually cause cell damage and dysfunction. In addition, subsidiary control of the adaptive responses of antioxidant enzymes to oxidative stress by Nrf2 in aging is another crucial factor that determines diminished antioxidant capacity in aging tissues [49]. Hence, antioxidants with the dual properties of prohibiting Nrf2 dysregulation and mitigation of NF-*κ*B may be useful for the protection of oxidative damage. It is possible that UCP modified early Nrf2 response to oxidative stress and maintained the whole antioxidant network homeostasis.

In summary, UCP is a dietary antioxidant that processes high antioxidant capacity and protects endothelial cells against H_2_O_2_-induced oxidative cell death mainly through lowering intracellular ROS, promotion of CAT activity, constraining overactivation of Nrf2, and reduction of NF-kB signaling. These multiple factors may forecast a superior protection of oxidative stress over small molecule antioxidant supplements mainly aiming for ROS scavenging activity as a single target. Nonetheless, research of using UCP as a dietary supplementation in the* in vivo* model of CVD and aging is warranted.

## Figures and Tables

**Table 1:** The regression equation of antioxidant potential of UCP.

Antioxidant capacity	(*μ*mol/g)	Linear Regression Equation

FRAP value	26.89 ± 1.14	y = 0.0234x + 5.140
ORAC (trolox equivalent)	56.03 ± 7.52	y = 0.0470x + 6.131

ROS Scavenging activity	IC_50_ (*μ*g/mL)	

Hydroxyl radical (OH^•^)	280.17 ± 3.57	y = 0.114x + 18.060
Hypochlorous acid (HOCl)	1316.13 ± 21.78	y = 0.036x + 2.951
Superoxide anion (O_2_^•–^)	846.53 ± 40.16	y = 0.317x - 12.470
Hydrogen peroxide (H_2_O_2_)	196.39 ± 7.36	y = 0.057x + 2.536

## Data Availability

The data used to support the findings of this study are included within the article.

## References

[1] Panieri E., Santoro M. M. (2015). ROS signaling and redox biology in endothelial cells. *Cellular and Molecular Life Sciences*.

[2] Daiber A., Steven S., Weber A. (2016). Targeting vascular (endothelial) dysfunction. *British Journal of Pharmacology*.

[3] Matsuzawa Y., Sugiyama S., Sumida H. (2013). Peripheral endothelial function and cardiovascular events in high-risk patients. *Journal of the American Heart Association*.

[4] Leopold J. A. (2015). Antioxidants and coronary artery disease. *Coronary Artery Disease*.

[5] Wang Y., Chun O. K., Song W. O. (2013). Plasma and dietary antioxidant status as cardiovascular disease risk factors: a review of human studies. *Nutrients*.

[6] Chen B., Lu Y., Chen Y., Cheng J. (2015). The role of Nrf2 in oxidative stress-induced endothelial injuries. *Journal of Endocrinology*.

[7] Kris-Etherton P. M., Hecker K. D., Bonanome A., Coval S. M., Binkoski A. E., Hilpert K. F. (2002). Bioactive compounds in foods: their role in the prevention of cardiovascular disease and cancer. *American Journal of Medicine*.

[8] Praticò D. (2005). Antioxidants and endothelium protection. *Atherosclerosis*.

[9] Gomes W. F., França F. R., Denadai M. (2018). Effect of freeze- and spray-drying on physico-chemical characteristics, phenolic compounds and antioxidant activity of papaya pulp. *Journal of Food Science and Technology*.

[10] Asghar N., Naqvi S. A., Hussain Z. (2016). Compositional difference in antioxidant and antibacterial activity of all parts of the Carica papaya using different solvents. *Chemistry Central Journal*.

[11] Aravind G., Bhowmik S. D. D., Harish G. (2013). *Traditional and Medicinal Uses of Carica Papaya*.

[12] Andrews C. M., Wyne K., Svenson J. E. (2018). The Use of Traditional and Complementary Medicine for Diabetes in Rural Guatemala. *Journal of Health Care for the Poor and Underserved*.

[13] Abe R., Ohtani K. (2013). An ethnobotanical study of medicinal plants and traditional therapies on Batan Island, the Philippines. *Journal of Ethnopharmacology*.

[14] Nayak S. B., Pereira L. P., Maharaj D. (2007). Wound healing activity of Carica papaya L. experimentally induced diabetic rats. *Indian Journal of Experimental Biology (IJEB)*.

[15] Dawkins G., Hewitt H., Wint Y., Obiefuna P. C., Wint B. (2003). Antibacterial effects of Carica papaya fruit on common wound organisms. *West Indian Medical Journal*.

[16] Somanah J., Ramsaha S., Verma S. (2016). Fermented papaya preparation modulates the progression of N -methyl- N -nitrosourea induced hepatocellular carcinoma in Balb/c mice. *Life Sciences*.

[17] Somanah J., Aruoma O. I., Gunness T. K. (2012). Effects of a short term supplementation of a fermented papaya preparation on biomarkers of diabetes mellitus in a randomized Mauritian population. *Preventive Medicine*.

[18] Barbagallo M., Marotta F., Dominguez LJ. (2015). Oxidative stress in patients with Alzheimer's disease: effect of extracts of fermented papaya powder. *Mediators of Inflammation*.

[19] Benzie I. F. F., Strain J. J. (1996). The ferric reducing ability of plasma (FRAP) as a measure of 'antioxidant power': the FRAP assay. *Analytical Biochemistry*.

[20] Thaipong K., Boonprakob U., Crosby K., Cisneros-Zevallos L., Hawkins Byrne D. (2006). Comparison of ABTS, DPPH, FRAP, and ORAC assays for estimating antioxidant activity from guava fruit extracts. *Journal of Food Composition and Analysis*.

[21] Mandal S., Hazra B., Sarkar R., Biswas S., Mandal N. (2011). Assessment of the antioxidant and reactive oxygen species scavenging activity of methanolic extract of caesalpinia crista leaf. *Evidence-Based Complementary and Alternative Medicine*.

[22] Valentão P., Fernandes E., Carvalho F., Andrade P. B., Seabra R. M., Bastos M. L. (2003). Hydroxyl radical and hypochlorous acid scavenging activity of small centaury (*Centaurium erythraea*) infusion. A comparative study with green tea (*Camellia sinensis*). *Phytomedicine*.

[23] Weydert C. J., Cullen J. J. (2010). Measurement of superoxide dismutase, catalase and glutathione peroxidase in cultured cells and tissue. *Nature Protocols*.

[24] Paital Biswaranjan (2014). A Modified Fluorimetric Method for Determination of Hydrogen Peroxide Using Homovanillic Acid Oxidation Principle. *BioMed Research International*.

[25] Phowichit S., Kobayashi M., Fujinoya Y. (2016). Antiangiogenic Effects of VH02, a Novel Urea Derivative: In Vitro and in Vivo Studies. *Molecules*.

[26] Johansson L. H., Borg L. A. H. (1988). A spectrophotometric method for determination of catalase activity in small tissue samples. *Analytical Biochemistry*.

[27] Chularojmontri L., Gerdprasert O., Wattanapitayakul S. K. (2013). Pummelo Protects Doxorubicin-Induced Cardiac Cell Death by Reducing Oxidative Stress, Modifying Glutathione Transferase Expression, and Preventing Cellular Senescence. *Evidence-Based Complementary and Alternative Medicine*.

[28] Ray P. D., Huang B., Tsuji Y. (2012). Reactive oxygen species (ROS) homeostasis and redox regulation in cellular signaling. *Cellular Signalling*.

[29] Guzik T. J., Touyz R. M. (2017). Oxidative stress, inflammation, and vascular aging in hypertension. *Hypertension*.

[30] Dinh Q. N., Drummond G. R., Sobey C. G., Chrissobolis S. (2014). Roles of inflammation, oxidative stress, and vascular dysfunction in hypertension. *Biomed Research International*.

[31] Cai H. (2005). Hydrogen peroxide regulation of endothelial function: origins, mechanisms, and consequences. *Cardiovascular Research*.

[32] Prior R. L., Wu X., Schaich K. (2005). Standardized methods for the determination of antioxidant capacity and phenolics in foods and dietary supplements. *Journal of Agricultural and Food Chemistry*.

[33] Blando F., Calabriso N., Berland H. (2018). Radical Scavenging and Anti-Inflammatory Activities of Representative Anthocyanin Groupings from Pigment-Rich Fruits and Vegetables. *International Journal of Molecular Sciences*.

[34] Mancini F. R., Affret A., Dow C. (2018). Dietary antioxidant capacity and risk of type 2 diabetes in the large prospective E3N-EPIC cohort. *Diabetologia*.

[35] Schieber M., Chandel N. S. (2014). ROS function in redox signaling and oxidative stress. *Current Biology*.

[36] Vendrov A. E., Vendrov K. C., Smith A. (2015). NOX4 NADPH Oxidase-Dependent Mitochondrial Oxidative Stress in Aging-Associated Cardiovascular Disease. *Antioxidants & Redox Signaling*.

[37] Makino N., Mochizuki Y., Bannai S., Sugita Y. (1994). Kinetic studies on the removal of extracellular hydrogen peroxide by cultured fibroblasts. *Journal of Biological Chemistry*.

[38] Martins D., English A. M. (2014). Catalase activity is stimulated by H_2_O_2_ in rich culture medium and is required for H_2_O_2_ resistance and adaptation in yeast. *Redox Biology*.

[39] Giordano C. R., Roberts R., Krentz K. A. (2015). Catalase Therapy Corrects Oxidative Stress-Induced Pathophysiology in Incipient Diabetic Retinopathy. *Investigative Opthalmology & Visual Science*.

[40] Sullivan-Gunn M. J., Lewandowski P. A. (2013). Elevated hydrogen peroxide and decreased catalase and glutathione peroxidase protection are associated with aging sarcopenia. *BMC Geriatrics*.

[41] Gomes P., Simão S., Lemos V., Amaral J. S., Soares-da-Silva P. (2013). Loss of oxidative stress tolerance in hypertension is linked to reduced catalase activity and increased c-Jun NH2-terminal kinase activation. *Free Radical Biology & Medicine*.

[42] Escribano A., Amor M., Pastor S. (2015). Decreased glutathione and low catalase activity contribute to oxidative stress in children with *α*-1 antitrypsin deficiency. *Thorax*.

[43] Zhang J., Wang X., Vikash V., Ye Q., Wu D., Liu Y. (2016). ROS and ROS-mediated cellular signaling. *Oxidative Medicine and Cellular Longevity*.

[44] Ighodaro O. M., Akinloye O. A. (2017). First line defence antioxidants-superoxide dismutase (SOD), catalase (CAT) and glutathione peroxidase (GPX): Their fundamental role in the entire antioxidant defence grid. *Alexandria Journal of Medicine*.

[45] Espinosa-Diez C., Miguel V., Mennerich D. (2015). Antioxidant responses and cellular adjustments to oxidative stress. *Redox Biology*.

[46] Farías J., Molina V., Carrasco R. (2017). Antioxidant Therapeutic Strategies for Cardiovascular Conditions Associated with Oxidative Stress. *Nutrients*.

[47] Mota S. I., Costa R. O., Ferreira I. L. (2015). Oxidative stress involving changes in Nrf2 and ER stress in early stages of Alzheimer's disease. *Biochimica et Biophysica Acta (BBA) - Molecular Basis of Disease*.

[48] Jiang L. J., Zhang S. M., Li C. W., Tang J. Y., Che F. Y., Lu Y. C. (2017). Roles of the Nrf2/HO-1 pathway in the anti-oxidative stress response to ischemia-reperfusion brain injury in rats. *European Review for Medical and Pharmacological Sciences*.

[49] Zhang H., Davies K. J., Forman H. J. (2015). Oxidative stress response and Nrf2 signaling in aging. *Free Radical Biology & Medicine*.

